# Back to the Future of Metabolism—Advances in the Discovery and Characterization of Unknown Biocatalytic Functions and Pathways

**DOI:** 10.3390/life14030364

**Published:** 2024-03-10

**Authors:** Roland Wohlgemuth

**Affiliations:** 1 MITR, Institute of Applied Radiation Chemistry, Faculty of Chemistry, Lodz University of Technology, Zeromskiego Street 116, 90-924 Lodz, Poland; roland.wohlgemuth.1@p.lodz.pl; 2 Swiss Coordination Committee Biotechnology (SKB), 8021 Zurich, Switzerland; 3 European Society of Applied Biocatalysis (ESAB), 1000 Brussels, Belgium

**Keywords:** enzyme functions, domains of unknown functions, central metabolic pathways, biosynthesis, biosynthetic gene clusters, cryptic pathways, orphan pathways, silent pathways, metabolite repair enzymes

## Abstract

The architecture, organization, and functioning of biocatalytic reaction networks, which are coded in the cell-specific genome and which work together in the small space of biological cells, are a fascinating feature of life evolved over more than 3 billion years. Knowledge about the diversity of biocatalytic functions and metabolic pathways sustaining life on our planet is highly important, especially as the currently occurring loss of biodiversity is considered a planetary boundary that is at high risk, and knowledge about the life of current biological organisms should be gained before they become extinct. In addition to the well-known enzymatic reactions involved in biochemical pathways, the enzyme universe offers numerous opportunities for discovering novel functions and pathways. Maintaining thousands of molecules and reactions functioning properly within biological cells, which may be exposed to various kinds of external hazards, environmental stress, enzymatic side reactions, or non-enzymatic chemical reactions, is key for keeping cellular life healthy. This review aims to outline advances in assigning enzyme functions to protein sequences and the discovery of novel biocatalytic functions and pathways.

## 1. Introduction

The fundamental question of how life emerged on planet Earth, which is up to now the only one among the thousands of exoplanets discovered, has been accompanying mankind in belief and scientific reflection systems for millennia and remains unsolved. Even though it is unknown how life originated on early Earth and there are many conflicting models [1,2], the concept of a primordial soup by Alex Oparin [3] has provided inspiration to the question of what type of components would have been needed in such a prebiotic soup [4]. Key features of living biological cells such as metabolism and self-organization, which are characterized by a complex organization in space and time of biocatalytic reaction networks coded in the cell-specific genome, have evolved over more than 3 billion years, with evidence for the oldest bacterial life forms near ancient submarine hydrothermal vents [5]. Whatever origin of life model is favored, metabolism is a key part, and criteria have been proposed that must be satisfied by chemical reaction systems to be a simple metabolism able to support protocell growth and division [6]. It is of much interest which catalysts and non-enzymatic reaction networks of ancient metabolism made use of simple starting materials and energy sources [7,8,9], prior to enzymatic reactions catalyzed by proteins and RNA enzymes. The known enzyme functions and pathways, which have been discovered over more than a hundred years up to now, are already catalyzing a tremendous diversity of reactions, not only in central metabolism but also in more remote and specialized metabolism, such as natural product biosynthesis [10]. The universe of enzyme functions and pathways is, however, much bigger and is expanding with the investigations of enzymes and biosynthetic pathways in a growing number of microbes [11], plants [12], and animals [13].

The great advances in molecular biology and genetics, as well as the impressive scientific and technological developments and improvements in methods for (a) analyzing and sequencing DNA [14] and RNA [15] and (b) synthesizing DNA [16] and RNA [17,18], have also revitalized the old interest in the influence of non-genetic factors such as nutrition, environment, competition, and symbiosis on health and disease of living organisms. Structural protein data collections, such as UniProt with more than 227 million protein sequences [19], PDB with more than 180,000 three-dimensional structures of proteins [20], and the more than 200 million predicted protein structures [21], provide a tremendous structural knowledge base for protein science and are advancing at a fast pace. Keeping up the pace of correct and experimentally verified functional annotations of protein sequences is essential [22], and the discovery of novel biocatalytic functions is supported by a variety of methodologies and tools linking genomics and enzymology [23]. Advances in the analysis of the whole small molecular domain (metabolome) of biological cells by MS and NMR methods have significantly enlarged the tools for identifying protein functions [24]. The simultaneous analysis of known protein functions in a biological cell is attractive for accelerating and extending the collection of enzyme kinetics data. This has been shown for 132 *Escherichia coli* enzymes by extracting, from omics data, the maximum catalytic rate that has been observed for each of these enzymes inside cells [25]. The synthesis of the respective protein and the small molecules predicted as substrates are, however, essential for the elucidation of in vivo effects on enzyme kinetics, for experimentally verifying the discovery of a novel enzyme function, and for confirming an already known protein function.

Fundamental discoveries, new approaches, and instrumental advances have enabled classical molecular biology and classical metabolism to meet in a new integrated molecular view of life, where the molecular biology central dogma [26] regarding sequential information transfer and the utilized alphabet is still key and the entire small molecular world of a biological organism is now also considered an important part of true molecular biology [27,28,29,30,31]. The more than 55 natural metabolite-sensing riboswitches, which have been discovered to control transcription termination, translation initiation, and alternative splicing, demonstrate the importance of linking the monitoring of biologically relevant elements, electrons, and ions in the form of ligands to riboswitches with the gene expression control and the complex metabolic pathway regulation. The way in which riboswitches typically achieve this sensing and control is by forming a domain within the 5′ untranslated region of the mRNA, which is able to bind small molecules as ligands and partly overlaps with an expression platform. Expression control is achieved by having the riboswitch in a genetic “on mode” when no ligand is bound, and a genetic “off mode” when a ligand is bound [32]. Although more than 55 classes of riboswitches for common cofactors and nucleotide-based signaling molecules have already been discovered, many opportunities still exist for discovering novel riboswitch classes sensing important metabolites, such as fatty acids, lipids, terpenoids, or unmodified sugars [33].

The opportunity to switch metabolic pathways and the involved enzymes on or off according to whether biologically relevant small molecules are present or absent in the nutrition or the environment connects metabolism with molecular biology and can be realized by different mechanisms. Metabolic pathway and network regulation can be achieved at multiple levels, such as genetic regulation [34], activation of silent pathways [35], or metabolite–protein interactions [36]. Metabolic efficiency can be achieved by separating highly reactive metabolites from the intracellular space in a protein-bound form, such as γ-glutamyl phosphate bound in L-glutamine synthase [37]. The hypothesis of metabolite–enzyme coevolution is attractive for integrating the avoidance of reaction losses through toxic and inhibitory metabolites, the recruitment of enzymes, and the evolution of metabolic pathways and networks [38].

The proper functioning of metabolic reactions also needs damage control and repair systems for the components of living biological cells which may be exposed to various kinds of damaging influences, either by external hazards and environmental stress or by internal factors such as the working life span of an enzyme [39], side reactions due to additional non-native catalytic functions or additional non-native substrate acceptance of enzymes [40] or chemical reactions within biological cells, which can not only damage macromolecules like nucleic acids and proteins but also metabolites [41]. Repair systems for damaged metabolites, which may be just useless when having no biological effects but in more severe cases may have negative biological effects, are important for maintaining healthy life, and biocatalytic functions and pathways converting damaged metabolites back to useful and non-toxic pathway metabolites contribute to robustness and stability [42]. The analysis of a minimized Mycoplasma JCVI-Syn3A genome has shown that metabolite damage repair systems are still in place and cannot therefore be separated from life itself [43].

Metabolites also play key roles in regulatory processes by the enzymatic modifications of essential biopolymers of molecular biology, such as DNA, RNA, and proteins. The enzymatic modification of histones and DNA, which involves metabolites from nutrients and the microbiome, connects metabolism and epigenetics in a complex interplay [44]. Other important interfaces between metabolism and molecular biology appear in gene silencing [45], post-transcriptional RNA modification [46], and post-translational modifications of proteins [47].

Tremendous advances have been achieved in assigning enzyme functions to protein sequences and the discovery of novel biocatalytic functions and pathways. More than 6000 enzymes, which have been recognized and categorized according to the reaction they catalyze by a four-digit EC number within the seven EC classes [48], have been shown to already have a large variety of enzyme activities. As the universe of enzymes is, however, much larger, moving the frontiers of enzyme function knowledge is therefore highly important. This is clearly evident from the growing number of sequences and structures of known proteins, for which the corresponding enzyme function is unknown or has not been experimentally verified. The main aim of this work is to bring back attention to metabolism as a key research area for the study of life and as a valuable resource for applications in biocatalysis. The great progress in genomic enzymology tools and methodologies and the discovery of unknown biocatalytic functions and pathways, as well as experimental technologies for analysis and synthesis, enlarge the power and opportunities of biocatalysis [49].

In addition to biocatalysts having a single enzyme function, biocatalysts have been discovered that can catalyze more than one reaction. Biocatalysts have been discovered that can switch their enzyme function depending on the pH, such as the terpene cyclases AaTPS and FgGS, which can act as aromatic prenyltransferase for generating prenylindoles at a basic pH [50]. Biocatalysts containing multiple enzyme functions in the same protein are well known and of much interest for stabilizing labile intermediates and directing cascade reactions selectively towards the rapid generation of molecular complexity [51]. Multifunctional enzymes, which are among the largest and most complex enzyme machineries, are involved as megasynthases in catalyzing the biosynthesis of numerous product groups, such as fatty acids, non-ribosomal peptides, polyketides, or terpenes, from simple natural building blocks. The biosynthesis of a large diversity of complex polyketides is catalyzed by multifunctional polyketide synthases, which can operate by the programmed use of the same enzyme functions repeatedly in an iterative mode, or by a linear channeling of the intermediates from one function to the subsequent function in an assembly-line mode [52,53,54].

## 2. Discovery and Characterization of Proteins with Unknown Biocatalytic Functions

Life on planet Earth at this timepoint can be categorized into ancient living organisms which have become extinct now, the biological organisms living now, and the biological organisms evolving in the future. As the currently occurring loss of biodiversity is of major concern [55], it is of much interest to gain knowledge about the life of current biological organisms before they become extinct. From genes to proteins to metabolites, the characterization of hidden biocatalytic functions and pathways, whether cryptic or silent [56], is an exciting research area.

### 2.1. The Sequence-Function Gap

One key area for understanding the metabolism of a biological organism in healthy and diseased conditions is knowledge about the sequence, structure, and function of their constituting proteins. The knowledge of gene sequences coding for proteins is growing much faster than the corresponding experimental identification and verification of their corresponding protein functions. This widens the gap, which represents a major challenge, as the assignment and experimental characterization of biocatalytic functions to the corresponding gene products requires substantial effort. The deep learning model DeepECtransformer has been developed to predict known enzyme functions, at the level of EC numbers, in order to reduce the number of un-annotated genes [57]. Enzyme functions, at the level of EC numbers, were predicted by DeepECtransformer from 464 un-annotated *E. coli* genes for the corresponding proteins, from which three proteins were randomly selected, the predicted glucose 1-dehydrogenase for YgfF, L-threonylcarbamoyladenylate synthase for YciO, and phosphonoacetate hydrolase for YjdM [57]. This facilitated experimental validation of the enzyme functions, which was performed by in vitro enzyme assays of the overexpressed and affinity-purified proteins YgfF, YciO, and YjdM [57]. For special enzyme reactions or completely novel enzyme functions without EC numbers, significant effort and time may be needed for the development of suitable analytical and preparative methods [22,58,59]. These include the expression and purification of proteins, synthesis of substrates, and analysis of substrates and products, as the catalytic function of a protein needs to be demonstrated by its incubation with a potential substrate and the identification of the nature of the product formed from the substrate in a protein- and time-dependent way. Further experimental characterization is of much interest and includes the identification of the optimum reaction conditions and the measurement of catalytic performance parameters such as *k*_cat_ and *K*_M_. The reporting of these functional datasets according to the STRENDA guidelines, which are recommended by an increasing number of journals, and their deposition in the STRENDA database provide a modular framework in the workflow of processing, storing, and retrieving an increasing number of enzyme function data [60]. The growing Pfam database of protein sequence families [61], which have been generated according to a significant degree of sequence similarity of protein domains, is of much interest for connecting to enzyme functions and the evolutionary history of proteins, and for guiding experiments. The activity of a known natural enzyme has also been a common starting point for engineering and evolving the properties of the enzyme towards the optimum performance of a desired biocatalytic reaction under defined reaction conditions with respect to catalytic efficiency, selectivity stability, or substrate scope [62].

### 2.2. Domains of Unknown Functions (DUFs)

Whole-genome sequencing of organisms has yielded a large dataset of genes that code for proteins whose function is completely unknown. The description of domains of unknown functions (DUFs) for uncharacterized protein families started in 1998 with the first two members DUF1 and DUF2 [63].

Since then, the DUFs, both in absolute numbers and as a percentage of all protein families, have continuously increased to more than 20% of all protein families over the years [64], reaching DUF6807 in release 35.0 of the Pfam database, which has now been integrated into the InterPro database [65]. Genomic enzymology web tools, such as sequence similarity networks, enzyme similarity tools, genome neighborhood tools, and taxonomy tools, enable the exploration of databases towards the in vitro characterization of the enzyme activities of uncharacterized proteins [23,66,67,68]. The following paragraphs will provide several examples of cases where different techniques were used to discover the functions of proteins containing DUFs.

**Figure 1:**
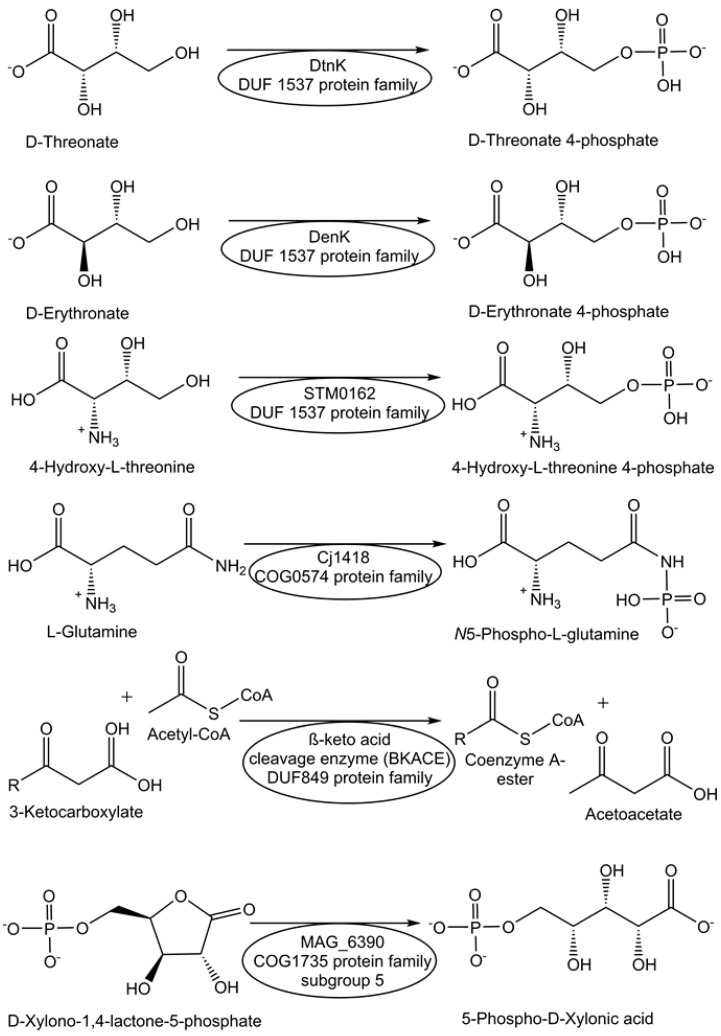
Assignment and characterization of enzyme functions for selected DUF proteins.

Screening of transport system proteins that bind solutes and applying sequence similarity networks and genome neighborhood networks have enabled the identification of novel kinases, which are ATP-dependent and act on four-carbon sugar acids, from the DUF1537 protein family [69]. Thereby, the novel DUF1537 enzymes D-threonate kinase DtnK and D-erythronate kinase DenK (see Figure 1) have been identified and characterized [69]. A strategy for finding enzyme activities within protein families of unknown function is based on defining a generic conserved reaction in the protein family, high-throughput screening, and analysis of genomic and metabolic context [69].

The protein Cj1418 from *Campylobacter jejuni*, which has been recombinantly expressed and affinity-purified, has been discovered as the first enzyme to directly phosphorylate the amide nitrogen [70]. Cj1418 has been clearly demonstrated to act as an ATP-dependent L-glutamine kinase (see Figure 1), which corrected its former annotation as a putative phosphoenolpyruvate synthase or pyruvate phosphate dikinase [70]. The application of this approach to the DUF849 Pfam family enabled the discovery of various novel β-keto acid cleavage enzymes [71].

For the functional annotation of the proteins Ms0025 from *Mycoplasma synoviae* and Mag6390 from *Mycoplasma agalactiae* (see Figure 1), for which no enzyme activity was known previously and no DUF number was reported, as novel lactonases, a combination of approaches was needed, from the consideration of genetic context, computational, empirical, and structural screening to the comparison of sequences and addition of newly synthesized substrates to the original libraries [72]. Both lactonases have been demonstrated to catalyze the hydrolysis of D-xylono-1,4-lactone-5-phosphate and the hydrolysis of L-arabino-1,4-lactone-5-phosphate [72].

## 3. Discovery and Characterization of Unknown Metabolic Pathways

Central metabolic cycles and pathways, such as glycolysis, mevalonate, and methyl-erythritol phosphate pathways, as well as the Calvin cycle, citric acid cycle, or urea cycle, have been discovered through significant scientific efforts and fundamental investigations, which have been honored by many Nobel Prizes and have become standard biochemistry knowledge. In addition to the central metabolic pathways for sustaining healthy life in the large diversity of biological species, specialized pathways for preparing bioactive small molecules from the nutrients available may be connected with special living conditions, diseases, or tasks. From microbes to plants, animals, and humans, the identification of the relevant biochemical pathways and missing enzymes continues to be highly important not only for natural and synthetic pathways to known bioactive metabolites and natural products but also for orphan, cryptic, or silent pathways to unknown metabolites, salvage, and repair pathways. Therefore, methods for identifying functional genes, such as gene expression profiling in real time, knockouts or heterologous expression of all the target genes of a complete biosynthetic pathway [73], combined analysis of genome and transcriptome data, and metabolome and enzymatic analysis, are essential for elucidating biochemical pathways [74]. This also requires outlining the organic chemistry of the biochemical pathways and connecting the metabolites with the genes that encode their biosynthesis [10,75]. Computational methods and tools using the databases of biochemical compounds and principles of biochemical reactions [76] are of much interest for potential unknown metabolic pathways towards shining light on the dark matter of metabolism.

### 3.1. Identification of Missing Enzymatic Reaction Steps in Metabolic Pathways

The identification of all enzymes and their functions along a biocatalytic pathway, as well as the metabolic intermediates, is key for a molecular understanding of the natural pathway and for designing synthetic pathways. The identification of missing enzymatic reaction steps in metabolic pathways has been a classical area in the discovery of new and now well-established metabolic pathways. Newly developed experimental tools and methods have, however, enabled fresh and straightforward approaches to identify missing enzymatic reaction steps, often leading to the discovery of entirely novel enzyme functions.

**Figure 2:**
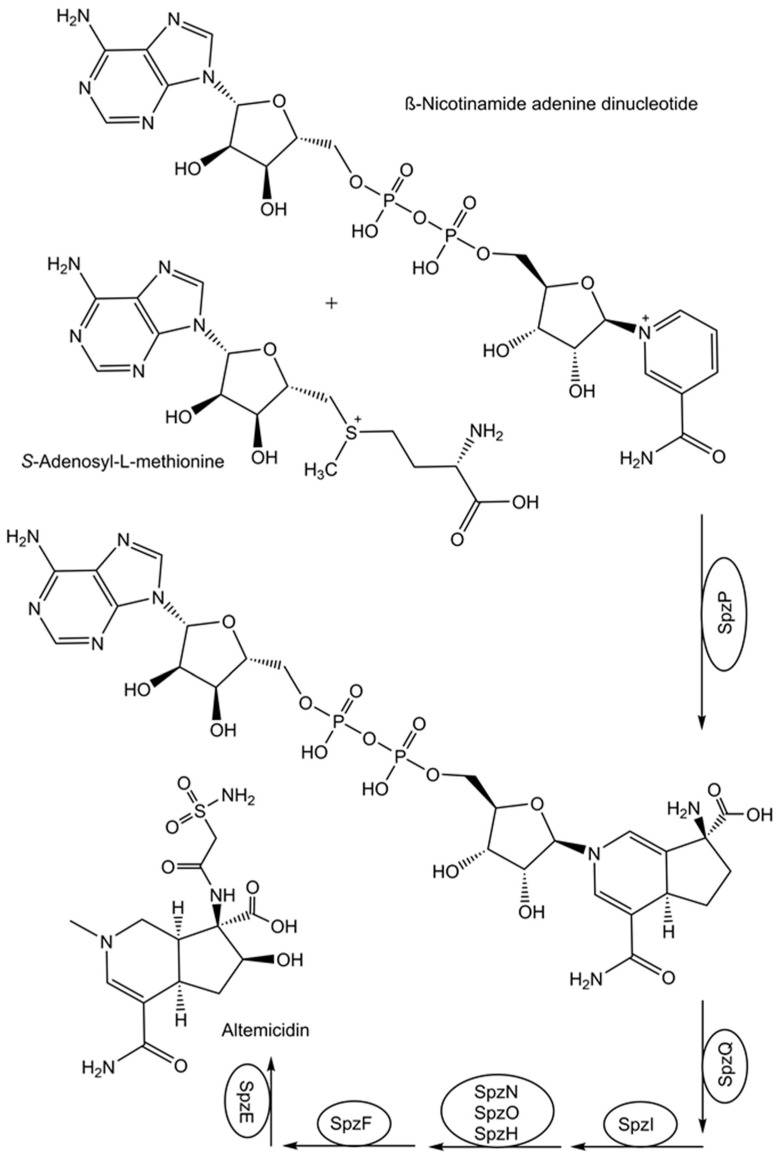
Identification of a missing enzymatic reaction step in the biosynthesis of altemicidin.

The question of how nature catalyzes the synthesis of altemicidin by the gene products of a recently identified biosynthetic gene cluster has been addressed by a smart combination of various experimental techniques [77]. This has led to the discovery of a fascinating novel pathway from β-nicotinamide adenine dinucleotide to altemicidin in eight enzymatic reaction steps (see Figure 2), whereby a novel enzymatic [3+2]-annulation between β-nicotinamide adenine dinucleotide and *S*-adenosyl-L-methionine was discovered [77]. From the separately expressed genes of the *Streptomyces lividans* biosynthetic gene cluster sbz and functional analysis by untargeted metabolomics analysis, SpzP has been found as the gatekeeping enzyme in the generation of the 6-azatetrahydroindane backbone [77].

The identification of all missing enzymatic reaction steps for completing the whole biosynthetic pathway of metabolites traditionally extracted in low yields from biological species is not only of fundamental interest but also provides a starting point for developing sustainable multi-step enzyme-catalyzed processes for their production. The identification of all missing enzymes in the complex 31-step vinblastin biosynthetic pathway of *Catharanthus roseus* has demonstrated how these enzymes catalyze the resource-efficient generation of chemical complexity from the simple metabolites tryptophan and geranylpyrophosphate by a combination of divergent and convergent synthesis strategies [78,79,80,81]. The question of how stemmadenine acetate, which is formed from strictosidine [79], is converted by divergent biocatalytic reactions to the two metabolite building blocks cataranthine and tabersonine has been addressed by chemical investigations, sequence data, gene silencing, synthesis of metabolite standards, NMR, and mass spectrometry [78]. These methods and the validation of the biocatalytic reaction steps *in vitro* with expressed and purified proteins enabled the discovery of two novel redox enzymes, which have been named precondylocarpine acetate synthase (PAS) and dihydroprecondylocarpine acetate synthase (DPAS), and the characterization of the two hydrolases tabersonine synthase (TS) and catharanthine synthase (CS) [78]. The two enzymes PAS and DPAS have been shown to catalyze the conversion of stemmadenine acetate into the unstable intermediates precondylocarpine acetate and dihydroprecondylocarpine acetate, which is converted by TS- or CS-catalyzed desacetoxylation to dehydrosecodine [78] to subsequently generate, through Diels–Alder cyclizations, either the TS-catalyzed reaction to tabersonine or the CS-catalyzed reaction to catharanthine [78]. After the biotransformation of tabersonine to vindoline, which is catalyzed by seven enzymes [80], the convergent synthesis of the anticancer natural product vinblastine in the biosynthetic pathway is completed by the condensation of catharanthine and vindoline [81]. The benefit of identifying all missing enzymes in a pathway has been demonstrated by the impressive achievement of engineering the thirty enzymes catalyzing the reactions to vindoline and catharanthine into yeast, using a chemical-coupling reaction in the final step to vinblastine [82].

### 3.2. Discovery and Characterization of Diverse Core Metabolic Pathways

It is also of much interest how the diversity of biological cells and their environments, from which the uptake of nutrients and energy is needed, is also reflected in a diverse core of metabolic pathways for the biosynthesis of the central molecules of life. As biological cells utilize riboswitches for sensing a variety of important metabolite levels and nutrients containing the essential elements of life, high-energy electron carriers and ions, elucidating riboswitch–ligand pairs and their associations with genes encoding proteins whose functions are unknown is of interest for the discovery of the corresponding metabolic or signaling pathways [32,33]. Investigating different domains of life for central metabolic pathways can provide not only insights into fundamental reactions, unusual pathways, and evolution but also valuable novel biocatalytic functions. A reversible reductive tricarboxylic acid cycle was discovered in the chemolithotrophic thermophile *Thermosulfidibacter takaii* by a combination of genomic, metabolomic, and enzymatic analysis [83]. The biodiversity and biosynthetic potential of the human gut microbiome have been demonstrated by the identification of 19,890 primary metabolic gene clusters in 4240 genomes, representing an important milestone for advancing the understanding of its role in human physiology [84]. While the biosynthetic pathway to the essential cofactor coenzyme A has been well established in bacteria and eukarya, it was only recently that the entire coenzyme biosynthetic pathway in archaea was experimentally validated and demonstrated to be different from bacteria and eukarya [85].

### 3.3. Discovery and Characterization of Diverse Specialized Metabolic Pathways

**Figure 3:**
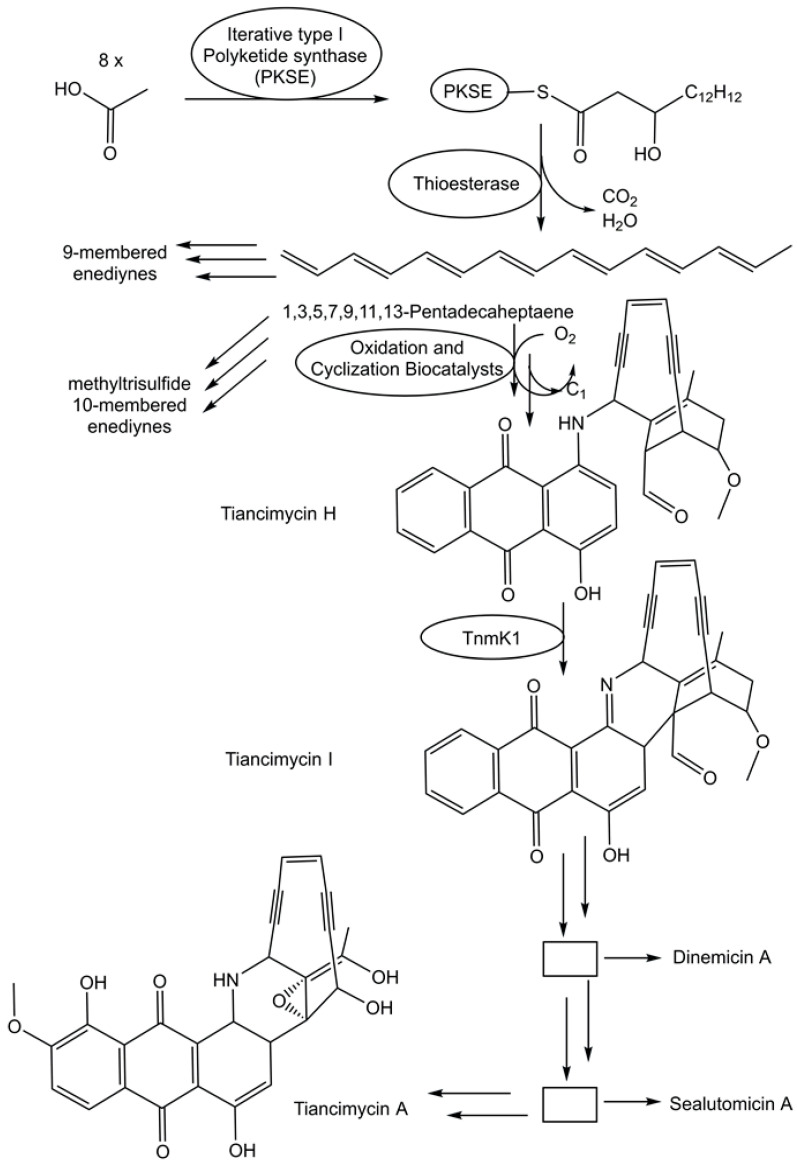
Proposed unifying pathway in the deciphering of the biosynthesis of the enediyne aromatic polyketides.

The fast growth of genome sequences in all domains of life has revealed the extent of unknown metabolic and biosynthetic capabilities of living organisms [86,87]. An impressive 231,534 biosynthetic gene cluster regions have been selected from archaeal, bacterial, and fungal genomes for the antiSMASH database version 4 [88]. Specialized metabolic pathways to complex and uniquely functionalized natural products therefore remain a very promising and vast area for discovering novel biosynthetic logic and biocatalytic functions. The biosynthetic pathway for enediyne aromatic polyketides has been investigated in recombinant *E. coli* strains. Combinations of genes that code for a polyketide synthase and a thioesterase from the enediyne biosynthetic gene clusters have been co-expressed in recombinant *E. coli* strains to complement mutant strains able to produce anthraquinone-fused enediynes but lack the corresponding polyketide synthase [89]. A combination of synthetic biology, chemical complementation, and ^13^C stable isotope labeling experiments enabled the identification of the common linear polyene intermediate 1,3,5,7,9,11,13-pentadecaheptaene and the proposal of a unifying pathway (see Figure 3) for enediyne aromatic polyketides [89]. This provides an excellent groundwork for the exploration of the intriguing biocatalytic reactions by which the pathways diverge from 1,3,5,7,9,11,13-pentadecaheptaene, whereby one molecule is transformed to the enediyne core, while the anthraquinone moiety is formed from a second one [89].

### 3.4. Discovery and Characterization of Hidden Metabolic Pathways

The search for various types of completely unknown or hidden biosynthetic pathways, such as cryptic, silent, or orphan pathways, leading to still unknown metabolites/natural products may not only provide novel biocatalytic functions and exciting new chemistry but also attractive novel scaffolds for biologically active small molecules [35,56]. A range of approaches have been developed for discovering novel structures of biologically active small molecules and for uncovering the links with the respective genes coding for the enzymes that catalyze the reactions leading to their biosynthesis [90]. Specialized biologically active small molecules may be only needed at specific times or certain conditions of life, and it is therefore not surprising that their biosynthesis is dependent on cultivation conditions, environmental signals, stress, presence of elicitors, inducers, or exogenous metabolites from co-cultivation [90]. These empirical approaches may, however, be challenging and impractical for larger numbers of samples. With the advances in genomics and the finding that, often, the genes that code for the enzymes used in a specific pathway are organized in biosynthetic gene clusters [91], genome-guided methods have attracted much interest [90]. Bioinformatics analysis tools for discovering biosynthetic pathways and for identifying biosynthetic gene clusters are very promising [92,93,94]. While genome sequences are important, much more is needed for connecting genes and the functions coded by them and for deciphering the biosynthetic logic of the corresponding metabolic pathways. Important complementary information can be derived from the combination of chemical and biochemical knowledge with genomic context [95], identification of co-expressed genes by RNA sequencing and transcriptome-wide analysis of differential gene expression [15], metabolomic analysis, and their correlation with absent or present expression of a biosynthetic gene cluster [96]. Finally, the integration of powerful analytical technologies with high information content is key for establishing the sequence of the biocatalytic reactions along the pathway and the molecular structure of the metabolites and natural products [97,98].

Bacterial and fungal genomes also have numerous silent biosynthetic gene clusters [86,87,88], which are poorly or not at all expressed under standard laboratory conditions. The question of how cryptic, orphan, and silent biosynthetic gene clusters, which outnumber the active ones, can be expressed is of fundamental importance for the discovery of unknown metabolic pathways. Therefore, much attention has been paid to general approaches and methods for activating and characterizing natural product biosynthetic routes as well as the corresponding genes encoding all the enzymes catalyzing the biosynthetic reactions. Various methods have been shown to be valuable, such as perturbation of epigenetic regulation, promoter exchange, control of the translation machinery by ribosome engineering, activator gene overexpression, or repressor gene inactivation [90]. The control of gene transcription by epigenetic regulation is of much interest for exploring the use of epigenetic modification for the activation of silent biosynthetic gene clusters [44,90]. The novel urea natural product class gaburedins was discovered via the derepression of its silent *gbnABC* biosynthetic gene cluster, which was achieved by deleting the putative regulatory gene *gbnR*, which is pathway-specific for the transcription repressor in *Streptomyces venezuelae* [99]. Structural determination of the metabolites which were present in the derepressed *gbnR* mutant but lacking in the silent wild type, the feeding of possible precursors, and the demonstration of no gaburedin biosynthesis when the *gbnB* gene was deleted in the *gbnR* mutant have led to the proposed roles of the enzymes GbnA and GbnB in gaburedin pathways [99]. The expression of silent biosynthetic genes was derepressed in a *Streptomyces* host by CRISPR/Cas9-mediated genome editing, after the capture of a specific *Streptomyces sclerotialus* biosynthetic gene cluster, which was cryptic and silent, and the transfer into the *Streptomyces* host [100]. A novel natural product class has been discovered by this approach, as demonstrated by (2-(benzoyloxy)acetyl)-L-proline, named scleric acid, and its proposed biosynthesis [100].

Other general approaches involve the insertion of promoters that are constitutively active using CRISPR-Cas9, identifying small molecules as inducers by high-throughput elicitor screening, and creating overproducing strains by reporter-guided mutant selection [101,102].

### 3.5. Discovery and Characterization of Salvage Pathways

Biological cells may have various provisioning paths to more complex metabolites and natural products in between their uptake from the environment and their complete biosynthesis from essential and simple low-molecular-weight biochemicals by natural or synthetic pathways, also termed de novo pathways. Nutrient supply issues may thereby be experienced at different levels, from intermediates and precursors to the more complex metabolites and natural products, in the uptake from the environment, or in the biochemical degradation of biopolymers. Therefore, the recycling and utilization of such intermediates and precursors by pathways to more complex metabolites and natural products, also termed salvage pathways, not only avoid the accumulation of waste and ensure resource efficiency but also support the life, resilience, and stability of biological cells, for example, by keeping adequate cofactor levels. Major coenzymes have been shown to be remarkably stable in vivo in *Escherichia coli*, *Bacillus subtilis*, and *Saccharomyces cerevisiae* [103].

The cofactor nicotinamide adenine dinucleotide (NAD^+^) with its de novo biosynthetic pathway from L-tryptophan via kynurenine is important for health and mitochondrial function [104]. The maintenance of adequate NAD^+^ levels is critical for a multitude of enzymatic and cellular functions, and several additional biosynthetic pathways to NAD^+^ have been discovered. NAD^+^ biosynthesis can be achieved in three enzymatic reaction steps (Preiss–Handler pathway) from niacin (nicotinic acid), whereby the last two steps converge with the kynurenine pathway [105,106,107]. Other biosynthetic pathways to NAD^+^ have been discovered, which start from the intermediates nicotinamide [108], nicotinamide riboside [109], nicotinic acid riboside [110] and its reduced form [111,112], and nicotinamide mononucleotide [113]. As these pathways require fewer reaction steps to NAD^+^, due to their use of metabolites containing the pyridine structure, and because they recycle these for the production of NAD^+^, a more inclusive use of the term salvage pathway seems reasonable [114,115].

**Figure 4:**
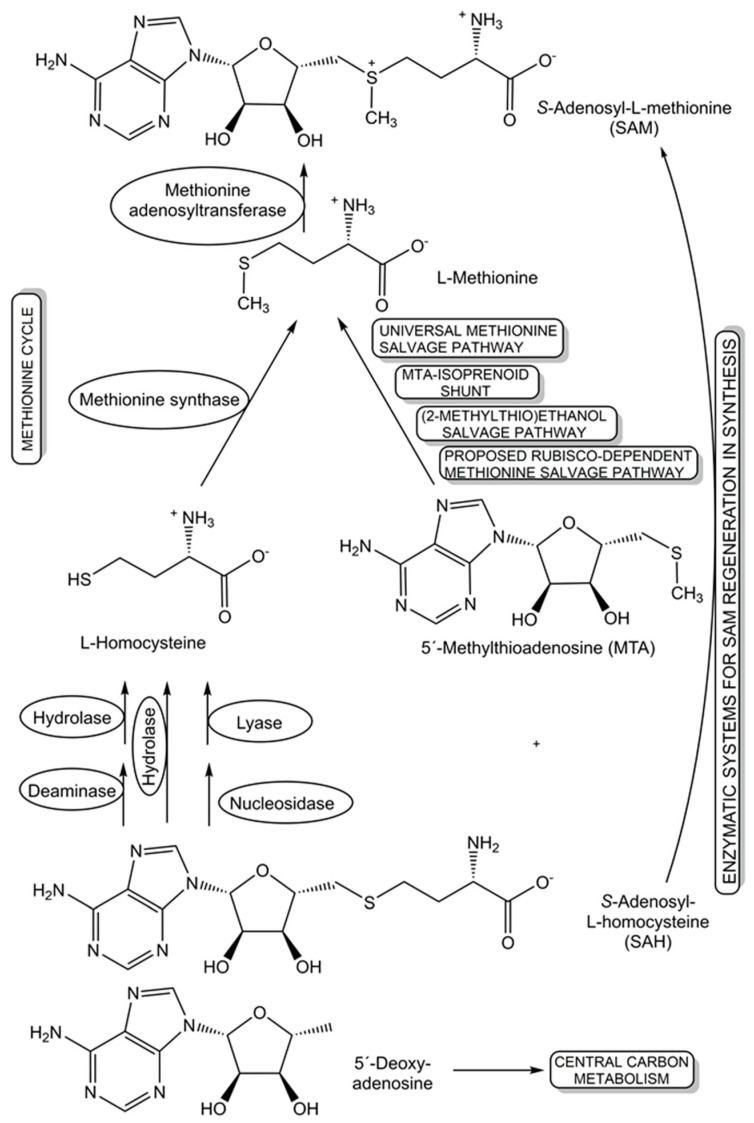
Diverse natural and synthetic salvage pathways that have been discovered, proposed, and designed for *S*-adenosyl-L-homocysteine, L-methionine, 5′-methylthioadenosine, and 5′-deoxy-adenosine.

The recycling and re-utilization of materials in salvage pathways provide biological cells with additional flexibility to maintain the levels of key cellular components, such as cofactors, under changing living conditions, while benefitting from shorter biosynthetic routes to complex cellular components. This re-utilization blueprint from nature is also of much interest for synthetic applications, for example in the recycling of cofactors. Further benefits of salvaging precursors have been demonstrated in populations of engineered *Escherichia coli* strains, where salvagers able to use the precursors cobinamide and 5,6-dimethylbenzimidazole for the biosynthesis of the complete cobamide vitamin B_12_ make this cofactor available to nonproducing consumer strains, are not overexploited, and remove nonfunctional and inhibiting precursors [116]. For the cofactor *S*-adenosyl-L-methionine (SAM), several salvage pathways for L-methionine and SAM byproducts are known, and two novel oxygen-independent salvage pathways for the SAM byproducts 5′-deoxyadenosine and 5′-methylthioadenosine have been discovered (see Figure 4) in *Rhodospirillum rubrum* and pathogenic *Escherichia coli* [117]. Salvage pathways leading back to SAM, either from nature or by design, can provide important tools for advancing and broadening the synthetic applications of different classes of SAM-dependent enzymes [118].

### 3.6. Discovery and Characterization of Biocatalytic Damage Control Systems

Genetically encoded biocatalytic systems for preventing the formation of damaged metabolites, or for repairing damaged metabolites and transforming them back into valuable metabolic intermediates, which cells can utilize again, are of key importance for maintaining the health of living cells. Biocatalytic damage control systems may be as important for cellular life over the course of time as biosynthetic pathways, as they can systematically counteract the negative effects of damaged metabolites, which can be formed from normal physiological metabolites by enzymatic side reactions or spontaneous chemical reactions to toxic products [41,42,43]. The enzymatic formation of damaged metabolites can occur under a variety of conditions, such as an unintended enzymatic transformation of a normal physiological metabolite or an enzymatic transformation of an unintended substrate in addition to the normal physiological substrate. Spontaneous chemical reactions occurring under physiological conditions have been assembled in the database CD-MINE, which is the abbreviation for Chemical Damage—Metabolic In Silico Network Expansion [119].

**Figure 5:**
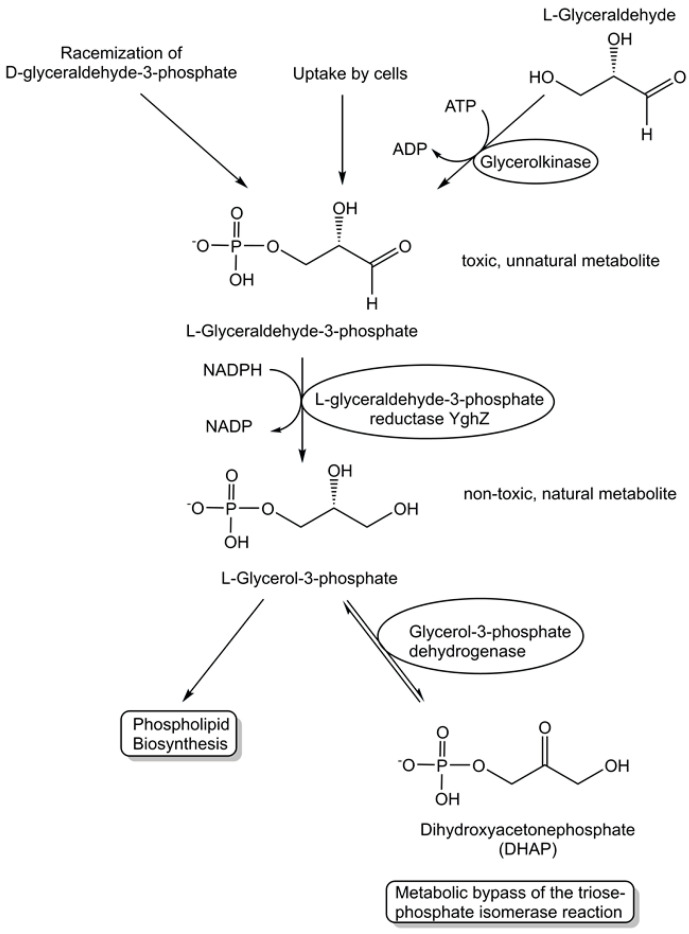
Discovery of the metabolite repair enzyme L-glyceraldehyde-3-phosphate reductase catalyzing the stereospecific, NADPH-dependent reduction of L-glyceraldehyde-3-phosphate to the non-toxic natural metabolite L-glycerol-3-phosphate (*sn*-glycerol-3-phosphate).

The activities of metabolite repair and clearance enzymes, which have been found to eliminate damaged metabolites and side-products in glycolysis [120], the citric acid cycle [121], photosynthesis [122], and other major pathways, are essential for the proper functioning of metabolic pathways. Damaged metabolites and side products that are toxic to biological cells have been a good starting point in the search for enzymes catalyzing their conversion to non-toxic natural metabolites that can be utilized in the corresponding cell metabolism. The repair of the intermediate 4-hydroxy-L-threonine, which is toxic, by its phosphorylation to 4-hydroxy-L-threonine-5-phosphate (see Figure 1), an essential metabolite of the pyridoxal-5-posphate pathway, has been demonstrated to be catalyzed by the kinase STM0162 (DUF1537) [123]. The unnatural metabolite L-glyceraldehyde 3-phosphate, which can be taken up by *E. coli* cells through different transport systems or formed by the glycerolkinase-catalyzed phosphorylation of L-glyceraldehyde or by a very slow non-enzymatic racemization of D-glyceraldehyde 3-phosphate [124], is toxic to biological cells due to its action as a bactericidal agent and enzyme inhibitor [125]. L-glyceraldehyde-3-phosphate reductase YghZ from *E. coli* has been discovered to be an enzyme for catalyzing the removal of toxic L-glyceraldehyde 3-phosphate by its conversion to L-glycerol-3-phosphate (see Figure 5), a natural non-toxic metabolite for use in phospholipid biosynthesis or for bypassing the triosephosphate isomerase-catalyzed reaction [125,126].

## 4. Discussion

The inventories of gene sequence information acquired from living organisms in all kingdoms of life, from genomes of uncultivated microorganisms as well as from metagenomes collected from different habitats, provide important resources [127,128,129] and document the genetic parts of life on planet Earth, even when biologically endangered species become extinct due to the ongoing loss of biodiversity. Whole-genome sequencing and the growth of gene sequence datasets are, however, much faster than the assignment of enzyme functions to the corresponding protein sequences. Therefore, the percentage of annotations with experimental evidence of even the ten most annotated genomes was found to vary from 2.5 to 21% in the year 2022 [130]. This sequence–function gap regarding genetically encoded biocatalytic functions is thus an exciting and fertile field for the discovery and characterization of novel sequence–function relationships. Great advances and novel approaches, methods, and tools [23,66,67,68] are very promising for reducing the number of un-annotated genes and facilitating the significant effort and time connected with the experimental validation of enzyme functions in annotated genes. Biological organisms have also been shown to possess numerous metabolic and biosynthetic capabilities, coded by clustered and non-clustered biosynthetic genes, for which the identification of all enzyme functions in a pathway is essential. The combination of various approaches for identifying the missing enzymatic reaction steps in a biosynthetic pathway can create a fertile research environment for discovering entirely novel biocatalytic functions. Not only is this highly important for delineating all the reaction types and the complete reaction architecture in the pathways to known bioactive small molecules but also for deciphering orphan, cryptic, or silent pathways to unknown metabolites. For keeping cellular life healthy over the course of time, biocatalytic housekeeping and maintenance systems are essential for repairing damage, clearing toxic side products, removing waste, and represent another highly important field for discovering novel biocatalytic functions and pathways. Elucidating the machinery to systematically prevent, repair, and overcome damage from enzymatic side reactions or spontaneous chemical reactions is not only of fundamental interest for life but has important implications in the context of diseases with corresponding damage profiles and therapeutic approaches in molecular medicine.

## 5. Outlook

Interactions between synthetic organic chemistry and synthetic blueprints of natural biosynthesis are of much interest in comparing reaction mechanisms and selectivities and controlling sensitive intermediates, for example, in the comparison of the biosynthesis [74] and the recent total synthesis of altemicidin [131]. While many compounds, such as altemicidin, have been discovered in nature before their total synthesis in the laboratory, the laboratory synthesis of a compound inspiring the discovery of this structure in nature, a coincidence of synthetic work and independent isolation from nature, or the synthesis of an anticipated natural product not yet confirmed by isolation, has been outlined as a very interesting explorative area for the collaboration of synthetic organic chemistry and natural biosynthesis [132]. This could not only be instrumental in the synthesis of potential substrates for as yet unknown enzyme functions but also in the discovery of completely novel reactions and whole biocatalytic pathways to these anticipated natural products.

The use of omics technologies and the integration of genome mining and metabolomics analysis [133], designated as metabologenomics, looks very promising for directing the discovery of the correct links between biosynthetic gene clusters, their encoded enzymes, and enzyme-catalyzed reactions, and the corresponding natural products, as recently shown by the identification of 21 natural products from Ascomycetes and their biosynthetic gene clusters [134].
